# Comparison of Structural Changes in Nodding Syndrome and Other Epilepsies Associated With *Onchocerca volvulus*

**DOI:** 10.1212/NXI.0000000000200074

**Published:** 2022-12-21

**Authors:** Rajarshi Mazumder, Samson Kamya Lubowa, Noriko Salamon, Nicholas J. Jackson, Michael Kawooya, Pamela Rosemary Akun, Ronald Anguzu, Rodney J. Ogwang, Joseph Kubofcik, Thomas Nutman, Kevin Marsh, Charles Newton, Angela Vincent, Richard Idro

**Affiliations:** From the Department of Neurology (R.M.), David Geffen School of Medicine at University of California Los Angeles; Kampala MRI Centre (S.K.L., M.K.), Uganda; Department of Radiological Sciences (N.S.), David Geffen School of Medicine, University of California Los Angeles, CA; Division of General Internal Medicine and Health Services Research (N.J.J.), David Geffen School of Medicine at UCLA; Centre of Tropical Neuroscience (P.R.A., R.A., R.I.), Kitgum Site, Uganda; Makerere University (R.A., R.J.O., R.I.), College of Health Sciences, Kampala, Uganda; Laboratory of Parasitic Diseases (J.K., T.N.), National Institutes of Health, Bethesda, MD; Centre for Tropical Medicine and Global Health (K.M., R.I.), Nuffield Department of Medicine, University of Oxford, United Kingdom; Department of Psychiatry (C.N.), University of Oxford, United Kingdom; and Nuffield Department of Clinical Neurosciences (A.V.), University of Oxford, United Kingdom.

## Abstract

**Background and Objective:**

Nodding syndrome (NS) is a unique childhood-onset epileptic disorder that occurs predominantly in several regions of sub-Saharan Africa. The disease has been associated with *Onchocerca volvulus (Ov)*–induced immune responses and possible cross-reactivity with host proteins. The aim of this study was to compare structural changes in the brain on MRI between NS and other forms of onchocerciasis-associated epilepsies (OAEs) and to relate structural changes to the Ov-induced immune responses and level of disability.

**Methods:**

Thirty-nine children with NS and 14 age-matched participants with other forms of OAE from an endemic region in Uganda underwent detailed clinical examination, serologic evaluation (including Ov-associated antibodies to Ov-16 and Hu-leiomodin-1) and quantitative volumetric analysis of brain MRIs (1.5 T scanner) using Neuroreader, a cloud-based software.

**Results:**

Cerebral and cerebellar atrophy were the predominant features in both NS and OAE. On quantitative volumetric analysis, participants with NS had larger ventricular volumes compared with participants with OAE, indicative of increased global cortical atrophy (p_corr_ = 0.036). Among children with NS, severe disability correlated with higher degree of atrophy in the gray matter volume (p_corr_ = 0.009) and cerebellar volume (p_corr_ = 0.009). NS cases had lower anti-Ov-16 IgG signal-to-noise ratios than the OAE cases (*p* < 0.01), but no difference in the levels of the Hu-leiomodin-1 antibodies (*p* = 0.64). The levels of Ov-associated antibodies did not relate to the degree of cerebral or cerebellar atrophy in either NS or OAE cases.

**Discussion:**

This is the first study to show that cerebral and cerebellar atrophy correlated with the severity of NS disability, providing an imaging marker for these endemic epileptic disorders that until now have remained poorly characterized. Both NS and OAE have cerebral and cerebellar atrophy, and the levels of Ov-associated antibodies do not seem to be related to the structural changes on MRI.

Nodding syndrome (NS), a unique childhood-onset epileptic disorder characterized by pathognomonic repetitive head drops, is largely found in regions of sub-Saharan Africa.^1,2^ Epidemiologic studies have found a consistent association with *Onchocerca volvulus*, the causative agent of onchocerciasis (“river blindness”) that infects millions of individuals worldwide.^3-5^ The parasite is also associated with other forms of epilepsy, known as onchocerciasis-associated epilepsy (OAE).^6-9^

Although the results have been inconsistent,^10^ parasite-driven antibody responses cross-reacting with human leiomodin-1 protein or other proteins have been proposed to underly the epileptogenesis in NS.^11^ Leiomodin-1 protein is a member of the tropomodulin family and functions as an actin nucleating protein in smooth muscle cells,^12^ but it is also expressed in the mouse hippocampus, cerebellar Purkinje cells, and cortical neurons and on the membranes of newly formed neurons and astrocytes.^12^ Moreover, in vitro studies found antibodies to leiomodin-1 to be neurotoxic.^11^

The seizures in NS are characterized by the pathognomonic repetitive head drops or head nodding and progressive cognitive and functional decline, whereas patients with OAE generally experience generalized tonic-clonic seizures and have less severe functional or cognitive impairments. Thus, one might expect significant structural differences in the brain structures, but to date, there are few imaging studies in either condition. A study from Uganda included individuals with only NS, whereas both persons with NS and other epilepsies were evaluated in a Tanzanian study.^13,14^ In addition, recently electroclinical and qualitative imaging changes were reported in OAE in northern Uganda.^15^ These studies demonstrated diffuse cerebral and cerebellar atrophy in NS and other forms of epilepsies within the *Onchocerca volvulus* (Ov) endemic region.

Although there are overlapping pathologic features between NS and OAE, it remains unknown if there are specific features, e.g., patterns of brain atrophy, that could distinguish NS from OAE. Limited neuropathologic studies have identified global cerebral and cerebellar atrophy on gross macroscopic examination in NS, with some evidence of inflammatory changes and tau protein accumulation.^15,16^

Given the previously reported cerebral atrophy, we used volumetric quantitation by MRI to evaluate the differences in the structural changes on MRI associated with NS and compare with other forms of OAE. A clinical and radiologic comparison of NS and OAE may help us to further understand the pathogenesis of the 2 disorders, identify new markers of disease severity and functional impairment, and define more clearly if there is a role for Ov-related immunity.

## Methods

### Design, Setting, and Recruitment of Participants

This imaging study was nested in a larger case-control study (2016–2020) investigating the pathogenesis of NS in northern Uganda and its association with Ov-cross-reacting antibodies. A total of 154 patients with NS were consecutively recruited and compared with 154 controls with non-NS epilepsy and 154 healthy community children. For this study, NS cases and OAE cases were randomly selected to undergo MRI of the brain at Kampala MRI Centre, approximately 400 km away.

Thirty-nine NS cases met the following case definition: (1) head nodding with the age of onset of nodding ranging from 3 to 18 years and (2) at least one of the following minor criteria: (a) neurologic abnormalities (cognitive decline, other seizures, or neurologic abnormalities), (b) clustering in space or time with similar cases, (c) stunting or wasting, (d) delayed sexual or physical development, and e) behavior problems or other psychiatric symptoms. Sixteen OAE cases met the following criteria^9^: (1) epilepsy with seizure onset between the ages of 3 and 18 years, (2) no reported developmental difficulties before the onset of epilepsy, (3) no known risk for symptomatic epilepsy on history or clinical examination (i.e., history of perinatal injury, febrile seizures, stroke, meningoencephalitis, and cerebral malaria), (4) geographical clustering of other persons with epilepsy in the village, (5) never diagnosed with NS, and (6) positive test for anti Ov-16 serum IgG antibodies (confirming the exposure to *O. volvulus*).

**Figure 1:**
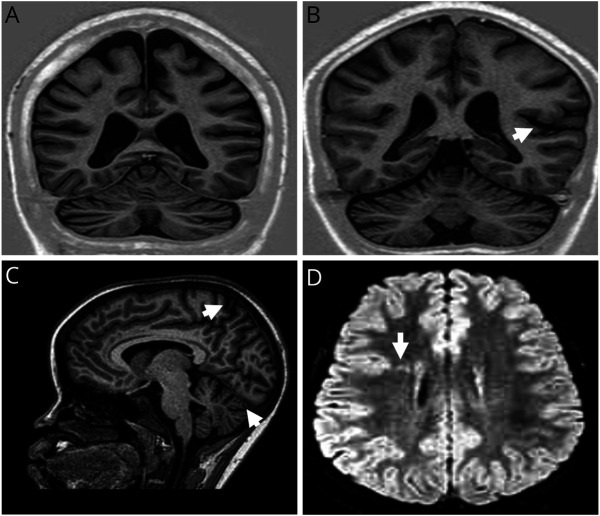
MRI of Participants With NS and OAE (A) 16-year-old girl with NS with global cerebral and cerebellar atrophy (T1- weighted inversion recovery). (B) 15-year-old boy with NS with left temporal lobe atrophy and bilateral cerebellar atrophy (white arrow showing the temporal lobe atrophy) (T1-weighted inversion recovery). (C) 14-year-old girl with OAE with cerebellar atrophy and global cerebral atrophy (small white arrow) (T1-weighted). (D) 17-year-old girl with OAE with type 2A focal cortical dysplasia in the inferior right frontal lobe (white arrow) (T2-FLAIR). Abbreviations: FLAIR = fluid-attenuated inversion recovery; NS = nodding syndrome; OAE = onchocerciasis-associated epilepsy.

MRI evaluation revealed developmental malformations in 2 of the 16 participants who met the clinical inclusion criteria for OAE (Figure 1 and Table 2). Given developmental malformations are a known cause of epilepsy, these 2 cases were removed from further quantitative imaging analyses.

### Antibody Testing

Exposure to *O. volvulus* was assessed by testing anti-Ov-16 IgG levels in plasma as previously described,^16^ with cut-offs based on standardized receiver operating curves (ROCs). In addition, anti-Hu leiomodin-1 antibody, the putative pathogenic antibody implicated in pathogenesis of NS, levels were evaluated using a modified LIPS assay as previously described^16^ using human leiomodin-1 fused to renilla luciferase as the “antigen.” ROC curves were again used to determine cut-offs; a sample was considered seropositive if signal-to-noise ratio was above a cut-off of 2 allowing for >99% specificity.

### Clinical Examination

All the participants underwent a standardized clinical assessment, which included detailed neurologic examination assessing mental status, cranial nerves, muscle strength, reflexes, sensory status, coordination, and gait. Nutritional status was assessed against WHO 2000 anthropometric standards. Functional status was assessed using the modified Rankin scale (mRS).^17^

### MR Acquisition

A 1.5 T (Achieva, Philips Medical Systems, The Netherlands) was used for all subjects. Anatomical MR imaging included pregadolinium and postgadolinium contrast T1-weighted images (particularly, sagittal 3D T1-weight sequences with 1.5 mm and no interslice gap yielding 175 slices), T2-weighted images, fluid-attenuated inversion recovery images, diffusion-weighted images, and susceptibility-weighted images. T1-weighted 3D volumetric scan included the following parameters: acquisition (ACQ) matrix = 180 × 239, ACQ voxel MPS (mm) = 1.11/1.23/1.11, repetition time/echo time (ms) = 7.0/3.2, A TI delay = 868.3, turbo field echo dur. shot/acq (ms) = 1,710.7/1,672.7, and flip angle (deg) = 8. The MRIs were also qualitatively evaluated by an experienced neuroradiologist (N.S.) blinded to the clinical status of the patient.

### MRI Processing

Automated volumetry was performed with a commercially available software program Neuroreader software (Brainreader Aps, Horsens, Denmark; brainreader.net/).^18^ This program, which performs automated volumetric segmentation, has been approved by the US Food and Drug Administration for automatic labeling, visualization, and volumetric quantification of MR images. We evaluated the volumes of the following region of interests: total brain, total white matter, total gray matter, total CSF volume, brainstem, lateral ventricles, frontal lobe, parietal lobe, occipital lobe, temporal lobe, hippocampus, amygdala, cerebellum, and the subcortical structures: caudate, putamen, thalamus (eFigure 1, links.lww.com/NXI/A789). The total intracranial volume was measured using volumes of white matter + gray matter + CSF + dura. The volume of the regions of interest were then normalized as the volume of region of interest/total intracranial volume × 100. This method has been consistently used to reduce the interindividual variability.^19^

### Statistical Analysis

Descriptive statistics (e.g., mean, SD, and relative frequency) were used to describe study variables for those with OAE and NS. Unadjusted between group differences were assessed using Student's *t*-test, Wilcoxon rank-sum test, or Fisher exact test as appropriate. Between group differences adjusting for the duration of disease and sex were conducted using linear regression. To understand the relationship between functional disability and the structural changes of the brain, the Pearson correlation was used for unadjusted analyses. Partial correlations were used to describe associations adjusted for sex and duration of disease. A Benjamini-Hochberg false discovery rate correction was used to adjust the significance threshold when examining multiple MRI structural measures in relation to functional disability. Analyses and graphs were created using Stata version 16.1, StataCorp LP (College Station, TX).

### Standard Protocol Approvals, Registrations, and Patient Consents

Approval for the study were obtained from the Makerere University School of Medicine Research and Ethics Committee (REC Ref: 2015-146) and the University of Oxford Tropical Research Ethics Committee (Ref: 12–16) and The Uganda National Council for Science and Technology (Ref: HS-1991). All parents provided written consent and assent was sought from participants 8 years or older who did not have severe cognitive impairment.

### Data Availability

Deidentified anonymized data used in this study will be shared upon request from any qualified investigator.

## Results

### Participants

Thirty-nine participants with NS (aged 15.6 ± 2.5 years) and 16 participants with OAE (aged 15.8 ± 1.3 years) were studied. The average age of onset of seizures was lower in the patients with NS (NS: 7.3 ± 3.0; OAE: 10.8 ± 2.7 years; *p* = 0.0001). As expected, generalized tonic-clonic seizures were more common in OAE (87.5%, n = 14) than in NS (51.3%, n = 20, *p* = 0.015). Participants with NS were more likely to have an abnormal gait (*p* = 0.025) or appendicular ataxia (*p* = 0.046). On neurologic examination, participants with NS were also more likely to exhibit abnormal cerebellar findings such as abnormal heel-to-sheen (*p* = 0.025) and finger-to-nose (*p* = 0.046). Compared with participants with OAE, participants with NS were more likely to have higher mRS score (*p* = 0.01; Table 1).

**Table 1:**
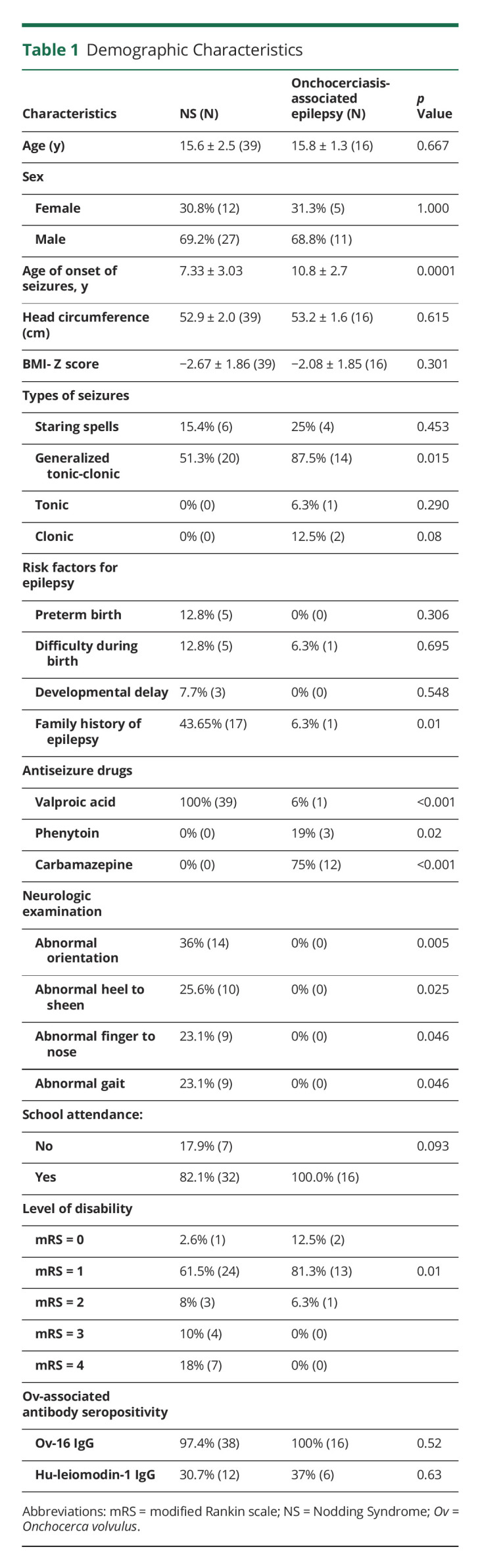
Demographic Characteristics

### Structural Changes in NS and OAE

Qualitative evaluation of MRI showed abnormalities in 31 of 39 (79.4%) participants with NS and 14 of 16 (87.5%) participants with OAE (Table 2). There was no enhancement noted with the administration of gadolinium contrast in any of the participants. Two participants (12.5%) with OAE had incidental findings of developmental malformations, particularly, focal cortical dysplasia and periventricular gray matter heterotopia (Figure 1). As noted earlier in the method section, these 2 participants were excluded from further quantitative imaging analysis. Global cerebral atrophy was present in 12.8% of NS and 6% of participants with OAE. Localized atrophy was more common in the parietal (NS: 17.9%; OAE: 6.2%), occipital (NS: 7.6%; OAE: 6.2%), or both parieto-occipital (NS: 15.3%; OAE: 25%) regions of both groups (Table 2). Cerebellar atrophy was common in both groups and was found in 28 (71%) patients with NS and 11 (68%) patients with OAE.

**Table 2:**
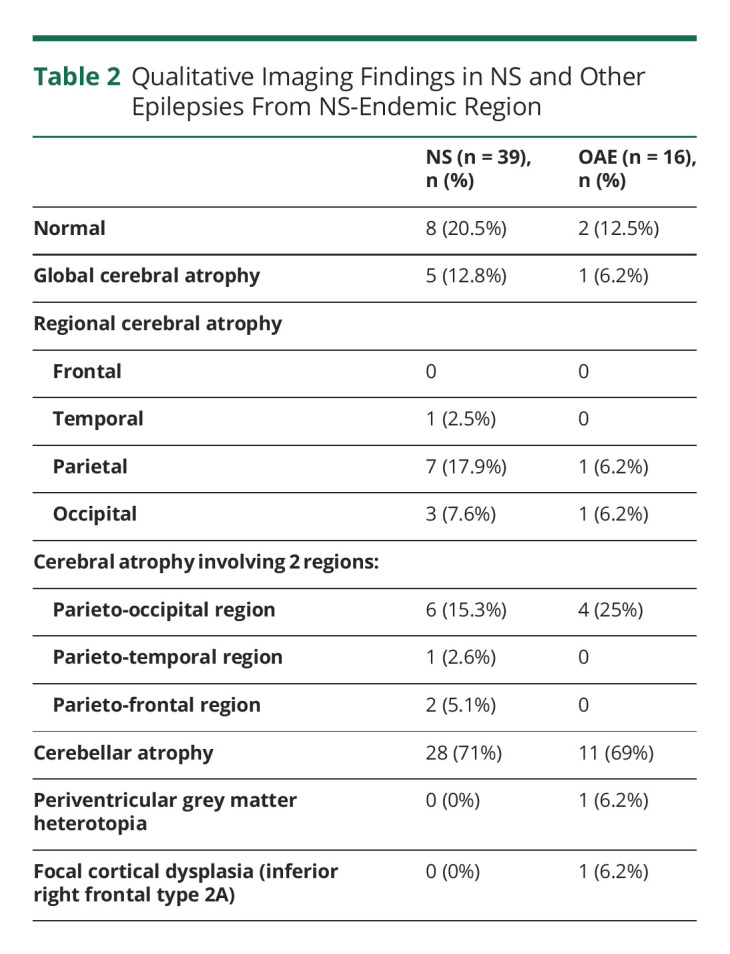
Qualitative Imaging Findings in NS and Other Epilepsies From NS-Endemic Region

**Figure 2:**
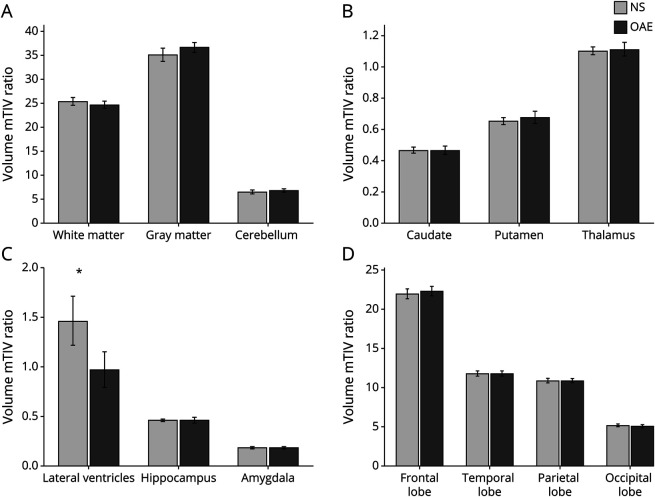
Quantitative Comparison of Regions of Interests Between NS (39 Cases) and OAE (14 Cases) Controlled for Duration of Seizures Global structures (panel A), subcortical structures (panel B), ventricle and mesial temporal structure (panel C), lobar regional structures (Panel D). Participants with NS were more likely to have larger ventricular volume compared to other epilepsies suggestive of increased global cortical atrophy (p_corr_ = 0.036). However, this difference was not statistically significant when controlled for duration of epilepsy (p_corr_ = 0.66). mTIV ratio = volume of region of interest/measured total intracranial volume X100. Abbreviations: NS = nodding syndrome; OAE = onchocerciasis-associated epilepsy.

On quantitative volumetric analysis of 39 NS and 14 OAE cases, participants with NS were more likely to have larger ventricular volumes compared with patients with OAE suggestive of increased global cortical atrophy (lateral ventricle/total intracranial volume ratio; NS = 1.461 ± 0.788 vs OAE = 0.962 ± 0.365; p_corr_ = 0.036). However, the difference in the ventricular volume was not statistically significant after adjusting for the duration of epilepsy (p_corr_ = 0.66; Figure 2). There was no significant volumetric difference between the NS and OAE in the regional cerebral structures or the cerebellum. Subcortical regions such as the caudate and the thalamus did not show any significant volume differences between the 2 groups (Figure 2).

### Hemispheric Asymmetry in NS and OAE

**Figure 3:**
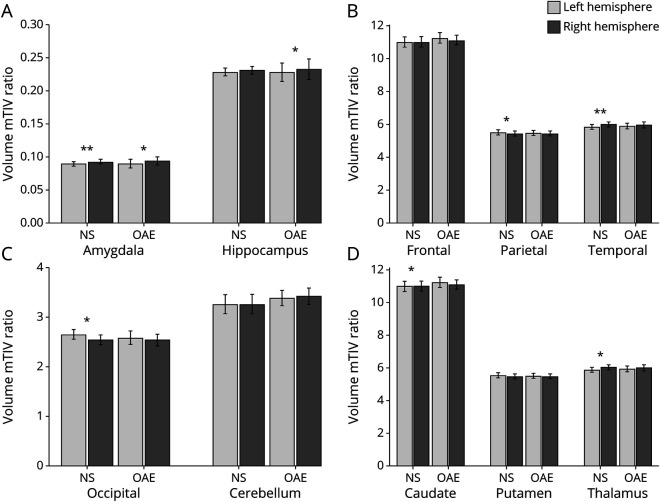
Regional Hemispheric Asymmetry in Participants With NS (39 Cases) and OAE (14 Cases) Medial Temporal structures that are highly vulnerable in epilepsy (Panel A); Regional lobar structures and cerebellum shows cerebral hemispheric asymmetry in NS (Panel B, C); Subcortical structures show hemispheric asymmetry of the thalamus and caudate in NS (Panel D). mTIV ratio = volume of region of interest/measured total intracranial volume X100. Abbreviations: NS = nodding syndrome; OAE = onchocerciasis-associated epilepsy

Among children with OAE, there was asymmetry in hippocampal volumes between the left and right hemisphere, not seen in children with NS, but both children with NS and OAE were noted to have asymmetry in the amygdala volumes. Hemispheric asymmetry was overall more common in participants with NS, noted in the subcortical regions, caudate, thalamus, lobar regions, parietal lobe, occipital, and temporal lobes (Figure 3).

### Brain Atrophy and Physical Examination in Children With NS

Among children with NS, individuals with abnormal gait were more likely to show volume loss after controlling for sex and age in the gray matter (p_corr_ = 0.038), brainstem (p_corr_ = 0.018), cerebellum (p_corr_ = 0.006), and several cerebral regions: frontal (p_corr_ = 0.006), parietal (p_corr_ = 0.018), and occipital (p_corr_ = 0.018). However, there was no association with volume loss of the temporal lobe or the subcortical structures: caudate (p_corr_ = 0.798), putamen (p_corr_ = 0.659), pallidum (p_corr_ = 0.798) or the thalamus (p_corr_ = 0.512) (eTable 1, links.lww.com/NXI/A789).

### Brain Atrophy and Measures of Disability Among Children With NS

**Figure 4:**
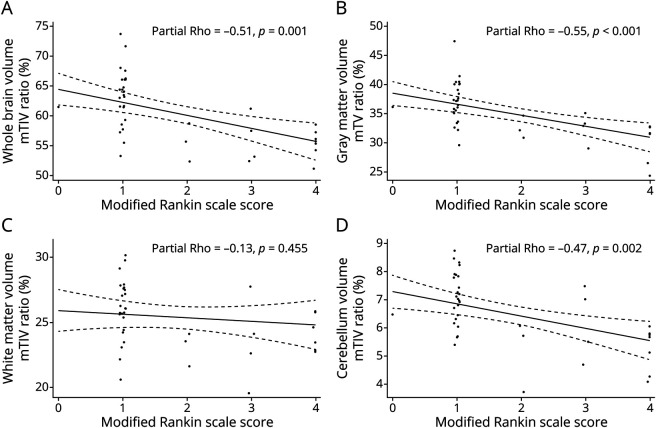
Association Between Regional Atrophy and NS, Adjusted for Sex and Duration of NS Increasing atrophy in the whole brain, gray matter and the cerebellum is associated with higher degree of functional disability (as noted by modified Rankin score). The relationship between white matter atrophy and functional disability is not statistically significant. mTIV ratio % = volume of region of interest/measured total intracranial volume X100. Abbreviation: NS = nodding syndrome.

Among children with NS after controlling for sex and duration of disease, higher mRS scores were correlated with a higher degree of atrophy in the whole brain volume (p_corr_ = 0.004), gray matter volume (p_corr_ = 0.004), and cerebellum (p_corr_ = 0.007) (Figure 4) but not with white matter volume or subcortical structures (Figure 4 and eTable 2, links.lww.com/NXI/A789). Increased cerebral regional atrophy also correlated with increased burden of disability in the frontal lobe (p_corr_ = 0.015), parietal lobe (p_corr_ = 0.004), and occipital lobe (p_corr_ = 0.004; eTable 2).

### Onchocerca-Associated Antibodies and Relationship With MRI

Overall, 97% of participants with NS and all (100%) of the participants with OAE were seropositive for Ov-16 IgG. Hu-leiomodin-1IgG was seropositive among 30.7% (n = 12), and 37% (n = 6) of participants with NS and participants with OAE, respectively (Table 1). There was no difference in the level of the Hu-leiomodin-1 antibody between the 2 groups (1.4 [range: 1.1–2.8] vs 1.6 [range: 1.3–2.2] *p* = 0.64), but NS cases had lower anti-Ov-16 IgG signal-to-noise ratio compared with the OAE cases (Ov-16 18.8 [IQR: 7.8, 36.7] vs 33.8 [IQR 25.6, 44.8], *p* < 0.01). There was no correlation between levels of Ov-associated antibodies (Ov-16 IgG, Hu-leiomodin-1) and atrophy in the regions of interest in either NS or OAE cases (eTables 3, 4, 5, 6, links.lww.com/NXI/A789).

## Discussion

NS and OAE are both thought to be onchocerciasis-associated immune-mediated epilepsy syndromes that commonly occur in Ov-endemic regions of sub-Saharan Africa, but it is unclear whether they are etiologically overlapping or distinct conditions. We used MRI combined with serologic and clinical data to study children with NS and OAE. Cerebral and cerebellar atrophy were common in both diseases. Although persons with NS had a higher degree of global cerebral atrophy, this difference was not statistically significant after adjusting for the duration of epilepsy. We also found that atrophy in both cerebellar and cerebral regions of interest correlated with the severity of disability in patients with NS. This is the first study to find these associations, and the results demonstrate the potential usefulness of MRI to explore the pathogenic mechanisms in NS and to compare further with OAE.

The overlapping features of cerebral and cerebellar atrophy on MRI between the 2 entities suggests that NS possibly represents a more severe form of OAE developing in younger children, as was previously proposed.^20^ Moreover, a quantitative volumetric analysis found amygdala asymmetry among both participants with OAE and NS. The amygdala may serve as an important structure in antibody-mediated epileptogenesis because damage to it has been seen in previous studies of other antibody-related limbic encephalitis.^21^

In other respects, the patterns of hemispheric asymmetry were mostly distinct between NS and the OAE cases. Hemispheric asymmetry was more widespread among individuals with NS and involved multiple subcortical (thalamus and caudate) and lobar regions. However, hippocampal asymmetry was only seen among cases of OAE and not among participants with NS. These differences in the hemispheric asymmetry between the 2 groups would argue against NS being part of the OAE spectrum disorder. The structural differences might suggest difference in the underlying differential connectivity of the brain regions in the 2 conditions and reflect the difference in seizure semiology, functional status, and the natural history of the disease. Future studies are needed to assess whether there are patterns of differential progression of the brain atrophy in NS and OAE, as commonly seen in neurodegenerative diseases.

The higher ventricular volumes in NS were likely to reflect global cortical atrophy because there were no evident white matter changes. However, the increased global atrophy in NS reflects a longer duration of the disease because the difference in the ventricular volume was not statistically significant after adjusting for the duration of epilepsy. Increased ventricular size is commonly seen in neurodegenerative disorders such as Alzheimer disease, where the rate of ventricular volume change is significantly correlated with an increase in neurofibrillary tangles.^22^ The presence of neurofibrillary tangles and tau, hallmarks of Alzheimer disease, have been reported in the pathology of NS^23,24^ but also in 2 of 4 cases with OAE.^23^ Alternately, it is possible that the increased global atrophy in patients with NS could reflect tau-associated pathologic changes. Future longitudinal studies are needed to address this important distinction.

The findings of cerebral and cerebellar atrophy among NS and OAE in our study is consistent with the previous report from Tanzania.^13^ Unlike the previous pathologic reports from Uganda and the MRI study from Tanzania, we did not find any evidence of gliosis in either NS or OAE.^13,23^ On qualitative analysis, there was no enhancement noted with the administration of gadolinium contrast in any of the participants, either with NS or OAE. This suggests a lack of ongoing disruption of the blood–brain barrier and inflammatory changes. The MRI findings of our studies are also congruent with histologic findings from postmortem studies in NS and OAE from Uganda, where cortical neuronal loss in the cerebrum and atrophy of the cerebellum is noted to be a prominent feature.^23,24^ Cerebellar degeneration because of Purkinje cell loss is common in several types of epilepsies and have been associated with the chronicity of seizures and the use of antiseizure medications such as phenytoin.^25-27^ In our study, only 19% of participants with OAE and none of the participants with NS were exposed to phenytoin. Thus, it is unlikely that drug exposure could have influenced the progressive cerebellar atrophy noted among the participants in both the groups.

Ov-associated antibodies (Hu-leiomodin-1 and Ov-16 IgG) were present in both participants with NS and OAE, with no statistically significant association between the level of antibodies and the degree of brain atrophy in either of the 2 groups. Although Hu-leiomodin-1 antibody is proposed to be a pathogenic immune mediator in NS by Johnson et al.,^11,12^ this association was not confirmed by Hotterbeekx et al.^10^ in a study with participants with OAE from DR Congo. There were significant differences in the methodology of these studies that could explain the difference in the findings. Although Johnson et al. included only participants with NS, Hotterbeekx et al. included persons with OAE and only 16 of 54 (29.6%) of these participants had nodding seizures. Given the significant difference in the two-study population, it would be difficult to compare their findings and draw conclusion about the etiologies of these Ov-associated disorders that have different clinical presentation. Moreover, the study by Hotterbeekx lacked detailed phenotyping (EEG or standardized semiology assessment or imaging). In our study, we found a significant proportion of OAE cases (12.5%) had developmental abnormalities that could cause epilepsy. Including all subtypes of epilepsies without detailed classification could have resulted in misclassification bias, where a low diagnostic specificity often reduces the estimation of the strength of association and is biased toward the null. The presence of Ov-16 IgG is related to past exposure to the Ov parasite and was an inclusion criterion for the patients with OAE in our study. Participants with NS and OAE were evaluated on an average of 8 years and 5 years after the onset of symptoms, respectively, and it is possible that the level of antibody titers in the chronic stage does not reflect the severity of the disease. Moreover, our imaging study did not include any age- and sex-matched healthy controls who were positive for Ov-associated antibodies or individuals with epilepsy without Ov-associated antibodies. This limits our interpretation of how the Ov-related antibodies might influence the structural changes of the brain. In addition, with a cross-sectional study design, we are unable to establish the temporal relationship between exposure to Hu-leiomodin-1 and the onset of NS and OAE.

The lack of diagnostic specificity for NS and OAE is a limitation of our study. Inclusion criteria for our study included the WHO clinical case definition of NS and the current consensus case definition of OAE.^9^ It should be noted that the current clinical case definitions do not include imaging, genetics, or EEG diagnostic testing. In Table 1, we have identified the proportion of our study population who are affected by other known risk factors of epilepsies. However, it remains unknown whether family history (genetic predisposition) and preterm birth are causally associated with NS. A total of 12.5% of OAE cases who met the inclusion criteria for our study were found to have developmental malformations. Our study underscores the role of neuroimaging, without which epilepsies due to structural abnormalities could be misclassified as OAE.

Nevertheless, we conducted a case-control study among a remote hard-to-reach population, some with severe disabilities, at an imaging center 400 km away from their villages. Standardized case definition was used to select cases and control-epilepsy participants that adds to the strength of the study. Our study is generalizable because the participants are from an area with a high burden of onchocerciasis reflecting other similar endemic regions where NS and OAE is highly prevalent, but prospective recruitment and study nearer to the onset of disease would be important in future studies.

## Glossary

mRS: modified Rankin scale
NS: nodding syndrome
OAE: onchocerciasis-associated epilepsy
*Ov*: *Onchocerca volvulus*
